# Indian – American contributions to psychiatric research

**DOI:** 10.4103/0019-5545.69209

**Published:** 2010-01

**Authors:** Anand K. Pandurangi

**Affiliations:** Virginia Commonwealth University, Richmond, Virginia, USA

**Keywords:** Indian – American, Indian Diaspora, Indian – American Psychiatric Research

## Abstract

The Indian Diaspora, especially in North America, is a visible force in the field of psychiatric medicine. An estimated 5000 persons of Indian origin practice psychiatry in the USA and Canada, and an estimated 10% of these are in academic psychiatry. Wide ranging contributions, from molecular biology of psychiatric disorders to community and cultural psychiatry, are being made by this vibrant group of researchers. This article is a brief summary and work-in-progress report of the contributions by Indian – American psychiatric researchers. Although not exhaustive in coverage, it is meant to give the reader an overview of the contributions made by three waves of researchers over a span of 50 years.

## INTRODUCTION

The Indian diaspora that has migrated to North America over the last 50 years, from 1960 until now, has established itself as a formidable intellectual force in Science, Engineering, Technology, Medicine, Education, Literature, and other fields.[1] Psychiatric Medicine has benefited from sustained and outstanding contributions of many academicians of Indian origin during this time. In this brief review, we present the contributions of this body of researchers, and make the first known attempt at tabulating Indo-American psychiatric researchers. In developing the data base for this article, several methods of data gathering were used, including searching through (1) Web pages and faculty rosters of major Departments of Psychiatry published on the web, (2) Google Scholar, (3) Forum[2] – a publication of the Indo-American Psychiatric Association, and (4) a brief survey of 25 known Indian psychiatric academicians, as well as the personal knowledge and observations of the author. We describe the researchers and their contributions in three chronological waves over the 50-year period.

### First wave (1960-1975)

The first wave of immigrants who pursued academic psychiatry arrived in the 1960s, to settle in the largest cities of Canada and the USA. This was the period when biological psychiatry and psychopharmacology were developing. Psychoanalytic psychiatry was on the wane and University Departments were transforming. Deinstitutionalization was in progress, community and social psychiatry were on the ascent, and biopsychosocial models of psychiatric disorders were being formulated. Foremost among this first wave of researchers was the Montreal group, which worked out of Douglas Hospital and Allan Memorial Institute of the Department of Psychiatry at McGill University. Jambur Ananth who had trained at the All India Institute of Mental Health, in Bangalore, (now called the National Institute of Mental Health and Neurosciences or NIMHANS) began his research career here in 1968. His collaborators included eminent researchers such as Samarthji Lal and NP Vasavan Nair. After a 13-year productive research career, Ananth moved to the University of California, Los Angeles in 1981. Ananth was a prodigious researcher and published over 400-articles, several books, and many book chapters. There are several themes to Ananth’s research, including, but not limited to, the biology and psychopharmacology of Schizophrenia and Depression. It would not be an exaggeration to say that until the year 2005, Ananth tested and helped develop virtually all psychopharmaceutical agents of the previous three decades. Noteworthy among his contributions are the role of nicotinic acid in psychoses, studies of the first and second generation antipsychotics, and antidepressants, and treatments for tardive dyskinesia. He edited the first text book on psychopharmacology authored predominantly by Indians.[3] Dr. N P Vasavan Nair and Dr. Samarthji Lal Ph. D, collaborators of Ananth, made significant independent contributions. Lal contributed heavily to our understanding of the role of monoamines in schizophrenia and depression, including the use of apomorphine in studying doapminergic function, and GABAergic regulation of dopaminergic neurons. He established a brain bank in Montreal. Vasavan Nair has conducted a career-long research into psychoneuroendocrinology, especially the relation between neurotransmitters, neuropeptides, and neurohormones, in the context of the biology of psychiatric disorders. He has published extensively on circadian biology and neurohormones, and more recently on the biology of aging. He established the Montreal Center for Studies on Aging.

In Chicago, Illinois, USA, another group of Indian researchers had formed in the late 1960s. Dr. Nedathur Narasimhachari, an organic chemist and faculty in the Department of Psychiatry at the University of Illinois and Illinois State Psychiatric Institute, worked closely with psychiatrists Harold Himwich and later John Davis. Their studies on the role of serotonin and its toxic variants in psychosis, and the presence of phenylethylamine in the human brain, are an important part of the history of biological psychiatry. Another stalwart psychiatric researcher from this institution, also not a psychiatrist is Ghanshyam Pandey. He is a professor of pharmacology in psychiatry at the University of Illinois Chicago and the Psychiatry Institute under the umbrella of the Illinois State Psychiatric Institute. Dr Pandey is a versatile researcher in many aspects of neurotransmitter function and is best known for his ground breaking research on the role of serotonin receptors in suicide. This institute has served as the formative and sustaining ground for many second-wave Indian psychiatrists and scientists, including psychiatrists Prakash Desai (Cultural and Social Psychiatry – see a little later in the text), Nagamani Pavaluri (Pediatric mood disorder), and Rajiv Sharma (neurochemistry of psychosis, gene regulation in neurons) who are all currently on its staff. Another pioneer from the ‘first wave’ with a lasting contribution to neuropsychiatric disorders is Dr. Harbans Lal, who promoted the concept of neuroprotection well before it became a fashionable term. Lal, a pharmacologist, held various teaching and research positions at the University of Kansas, University of Chicago, and University of Rhode Island, and eventually settled as Chair of Pharmacology and Neurosciences at the Medical College of the University of North Texas Health Science Center. Dr Sachin Pradhan, also a pharmacologist has contributed to our understanding of the effects of psychomimetic drugs such as PCP and inhalants. He has had a long tenure at Howard University in Washington DC, and edited a text book of pharmacology. Separately, he also published a book on the contribution of Indians in USA.[1] Dr. Salman Akhtar, psychiatrist, psychoanalyst and a postgraduate alumnus of PGIMER, Chandigarh, Department of Psychiatry, began his US career in 1972, and is one of the earlier members of this first wave of researchers and occupies a unique position among Indo-American scholars, as the undisputed leader in psychoanalytic psychiatry (see a little later in the text).

### Second Wave (1975-1990)

The second wave of researchers consist of those that began their research careers in USA or Canada between approximately 1985 and throughout the 1990s. By now, some of these academicians have nearly 30 years of contributions to their credit and occupy endowed chairs and leadership positions in academic psychiatry. Although no exact count exists, it is estimated that there are at least 50 such researchers forming a solid core of psychiatric academicians of Indian origin. Geographically, they have spread their wings across the span of USA and Canada, and are at leading academic centers of the two countries. This group has accumulated an impressive record of contributions from the bench to the remote corners of the community, and has deservedly acquired a reputation for intelligence, originality, diligence, collegiality, collaboration, and leadership, nationally and internationally. There are too many individuals in this cadre to mention all, and it is beyond the scope of this article to review their work individually. See Table 1for a selective listing of these individuals and their main areas (s) of work.

**Table 1:** 100 Indian–American psychiatrist researchers

Name	Area of work	Current affiliation
Adityanjee	Schizophrenia, NMS	U Minnesota
Amit Aiiand	Imaging. Mood disorders	Indiana U
Nutan Atre-Vaidya	Psychiatric education	Rosalind Franklin U, Chicago
Praful Chandaraiia	Psychiatry education Psychosomatics	U Western Ontario, Canada
Bhaskar Dave	Public psychiatry. Residency education	Independence MH Institute Iowa State
D Devanand	ECT, Memory disorders	Columbia U, NY
Murali Doraiswami	Biological psychiatry Brain imaging	Duke U. Durham, NC
Sanjay Dube	Psychopharmac ology. Drug development	Eli Lilly, Indianapolis
Prakash Ettigi	Mood disorders	VCU, Richmond, VA
Kishore Gadde	Obesity, weight loss pharmacology	Duke U. Durham, NC
Rohan Ganguli	Psychoimmunology, Psychopharmac ology	U Toronto and U Pittsburgh
Lilian Gonsalves	Pain, women’s health	Cleveland Clinic
Shiv Hatti	Clinical drug trials	Suburban Assoc Philadelphia
Geeta Jayaram	Quality assurance International service	Johns Hopkins, Baltimore, MD
Ripu Jindal	Psychopharmac ology	U Pittsburgh
Matcheri Keshavan	Schizophrenia, Early psychosis. Brain imaging	Harvard U, Boston and U Pitt, Pennsylvania
Ranga R Krishnan	Biology of depression. Brain imaging	Duke U, NC and Singapore NUS
Benji Kurian	Depression	UTSW, Texas
Sheila Loboprabhu	Geriatric psychiatry, End-of-life care	Baylor Col Med Houston. Texas
Atul Mahableshwarkar	Schizophrenia, Depression, PTSD	UIC, Chicago, IL
Ashok Malla	Early psychosis, schizophrenia	McGill U, Montreal, Canada
Mehul Mankad	Forensic psychiatry, ECT	Duke U. Durham, NC
Manu Matthews	Pain management, depression	Cleveland Clinic, Ohio
Asha Mishra	Community psychiatry, cultural psychiatry	VCU, Richmond. VA
Dinesh Mittal	Delirium, anxiety and depression	U Arkansas
Surinder Nand	Cultural psychiatry, psychiatric education	UIC, Chicago
Meera Narasimhan	Mood D/O, primary care and psychiatry, telepsychiatry	USC, Columbia. SC
Viswajit Ninigaonkar	Psychiatric genetics	U Pittsburgh
Prasad Padala	Alzheimer disease, PTSD	U Neb, Omaha. Nebraska
Anand Pandurangi	Schizophrenia, imaging psychopharmacology	VCU, Richmond. VA
Ashvin Patkar	Addiction psychiatry	Duke U, Durham, NC
Salman Akhtar	Psychoanalysis	Thomas Jefferson U Pennsylvania
Jambur Ananth**	Psychopharmacology, culture and psychiatry	UCLA, California
Subhash Bhatia	Addictions, geriatric psychiatry	Creighton U, Nebraska
Roy Chengappa	P syc hopharmac ology, bipolar disorders	U Pittsburgh
Prakash Desai	Bio ethics	UIC, Chicago
Mantosh Dewan	Psychiatry education psychotherapy, imaging	Upstate Med U, Syracuse, NY
Deepak D’Souza	Schizophrenia	Yale U, Connecticut
Shashi Elongovan	Child psychiatry	Long Island. NY
Ranga esh Gadasalli	Antipsychotics	UCLA, California
Mary Ganguli	Geriatric psychiatry, epidemiology	U Pittsburgh
Sanjay Gupta	Mood and memory disorders in elderly	U Buffalo
Chetan Haldipur	Psychiatry education History of psychiatry	Upstate Med U. Syracuse, NY
Choudhury Jampala	Schizophrenia	Rosalind Franklin U, Chicago
Dilip Jeste	Schizophrenia, TD, late life psychosis, nosology	UCSD, California
Nalini Juthani	Psychiatry education Cultural psychiatry	Albert Einstein Col Med, NY
Prasad Konsale	Imaging, genetics	U Pitt, Pittsburgh, PA
Anand Kumar	Geriatric psychiatry	UIC, Chicago
Vivek Kusumakar**	Bipolar disorders, child psychiatry	J andJ, NJ
Rajnish Mago	Mood disorders	Jefferson Med Col Philadelphia
Anil Malhotra	Genetics schizophrenia, pharmacogenetics	LIJ/Albert Einstein, NY
Rahul Manchanda	Early psychosis, neurorehabilitation	U Western Ontario, Canada
Prakash Masand	Psychosomatics, psychopharmacology	Duke U. Durham, NC
Sanjay Matthews	Biology and imaging in mood and anxiety D/O	Baylor Col Med, Houston. Texas
Shaila Misri	Women’s mental health	U BC, Vancouver, Canada
Sukdeb Mukherjee**	ECT, biology of schiz and bipolar D/O	Columbia U, NY
NPV Nair	Psycluieuroendocrinology	McGill U. Montreal
Nikhil Nihalam	Forensic psychiatry Psychosomatics	Behavioral Health Services. Valdosta. GA
Phillip Ninan	Anxiety and mood disorders	Pfizer Phamia. Philadelphia
Atul Pande	Psychopharmacology	GSK Durham, NC
Anand Pandya	Disaster psychiatry	UCLA Semel Institute
Nagamani Pavaluri	Pediatric mood D/O	UIC Chicago
Parameswaran S	NMS	UCLA Harborview
Rudra Prakash	Neuropsychiatry, personality disorders	Vanderbilt U, Nashville, TN
Jaisimha Rao	Developmental disorders	St Joseph’s Health Ontario, Canada
Uma Rao	Child psychiatry – mood D/O. addictions	UTSW Dallas, Tx
Ravinder Reddy	Schizophrenia, imaging	U Pittsburgh
B M Saxena	Schizophrenia, psychopharmacology	U Western Ontario Canada
Manoj Shah	Albert einstein coll, NY	Adolescent and Child Psychiatry
Rajiv Shanna	Biology of psychoses, gene regulation	UIC, Chicago
Ramakrishna Shenoy	Mental retardation and developmental D/O	VCU, Richmond. VA
Satish Shrikhande	Schizophrenia, psychopharmacology	UBC, Victoria, Canada
Jaskaran Singh	Psychopharmacology	Johnson and Johnson, NJ
Shamsah Sonawalla	Wromen’s mental health	Harvard U, Boston. Jaslok Hospital. India
Ashok Srinivasaragha Van	Forensic psychiatry	SIU Springfield, IL
Rajiv Tandon	Schizophrenia, psychopharmacology	UOF-Gainesville, FL
Harsh Trivedi	Public psychiatry	Cleveland, Ohio
Ipsit Vahia	Schizophrenia	UCSD California
Cherian Verghese	Clinical drug trials	Keystone Clinical Studies, Norristown, PA
R Vishwanathan	Addiction, anxiety, psychosomatics	SUNY Downstate. NY
Lakshmi Yatham	Bipolar disorders	U BC Vancouver. Canada
Haranath Parepally	Schizophrenia	U Pittsburgh
Rajaprabhakaran Rajaretinam	Schizophrenia, brain imaging	Wayne State U Detroit
Nyapati Rao	Geriatric psychiatry, psychiatry eeducation	SUNY Downstate. NY
Vani Rao	Brain injury	Johns Hopkins Baltimore, MD
Parikh V Sagar	Anxiety and mood disorders	University of Toronto, Canada
Vidyasagar Sethi	Molecular pharmacology	Carniel Psych Assoc Charlotte, NC
C M Shammi	Psychopharmac ology	U Toronto, Canada
Virender Sharnia	Depression	U Western Ontario, Canada
Shashi Shettar	Sleep disorders	U Mississippi Satish Shrikhande
Ram Shrivastav	Culture; psychopharmacology	Eastside Comp Med Ctr, NY
Nataraj Sitaram	Biology of sleep	Wayne State U Detroit
Aradhana B Sood	Child psychiatry	VCU, Richmond. VA
Shilpa Srinivasan	Geriatric psychiatry	U So Carolina Columbia. SC
Gunwant Thaker	Biology of schizophrenia	UMD, MPRC Baltimore, MD
Madhukar Trivedi	Depression, psychopharmacology	UTSW. Dallas, Texas
Ajay Vasan	Pain disorders	Harvard U, Boston
Sumer Vernia	Geriatric psychiatry	Harvard U, Boston
Seetharaman Vivek	Addictions, geriatric, and psychosomatics	Jamaica Hospital, NY
Vikram Yeragani	Anxiety disorders, heart rate variability	Wayne State U, Detroit and Nagarjun U, Vishakpatnam

** Deceased

Dr. Rohan and Dr. Mary Ganguli, Dr. Dilip Jeste, Dr. Matcheri Keshavan, Dr. Shitij Kapur, Dr. Ranga R Krishnan, Dr. Madhukar Trivedi and Dr. Lakshmi Yatham epitomize the cream of this crop. Jeste developed his research career at the National Institute of Health (USA) before moving to the University California at San Diego. The first phase of his research focused on the biology and treatment of schizophrenia, and subsequently on the identification and treatment of tardive dyskinesia (TD). TD had become a major challenge in the treatment of schizophrenia and it was important to know through systematic studies, its prevalence, cause, and potential treatment. Dr. Jeste rose up to this challenge and provided researchers and practitioners reliable data on the prevalence of TD over the short and long term. In the second phase of his research career, which started at UCSD, Dr. Jeste shifted his attention to geriatric psychiatry, and quickly assumed the mantle of an undisputed research leader in this field. Especially noteworthy is his work on psychoses in the elderly. Jeste also took on various leadership roles including being President of the Association of Geriatric Psychiatry and an editor of its flagship journal. Dr. Ranga Rama Krishnan of the Duke University is a prolific biological researcher of international fame. His group at Duke has conducted numerous investigations on the biology of depression, and championed the concept of vascular depression in the elderly, based on their original findings of excessive hyperintensity lesions demonstrated by magnetic resonance imaging. They are also considered leaders in the development of technology for psychiatric brain imaging. Ranga Krishnan became the first chair of Indian origin in a major private university such as Duke, and has gone on to International leadership in medical education by leading the development of the Duke Graduate Medical School in Singapore under the aegis of the Duke University and the National University of Singapore. Both Dr. Jeste and Dr. Krishnan were inducted into the Institute of Medicine, an honor bestowed on very select academicians. This speaks for the high stature they hold in the medical academia. Matcheri Keshavan is an alumni of NIMHANS, Bangalore. Dr. Keshavan received advanced research training in Vienna and worked at the Maudsley in London. In USA, his career began at Wayne State University, although the majority of his work occurred during his tenure at the Western Psychiatric Institute and Clinic (WPIC) of the University of Pittsburgh. Of late, he was appointed as an endowed professor and one of the Vice Chairs at the Harvard University. Keshavan’s contribution to the understanding of the first episode of psychoses, especially the integrity (or lack thereof) in the neuronal energy mechanisms, demonstrated by magnetic resonance spectroscopy is an excellent example of his incisive insights into the biology of psychosis. Another area of much clinical and biological research by Keshavan and his group has been in the neurodevelopmental origins of schizophrenia. The WPIC in Pittsburgh serves as the psychiatry department of the University of Pittsburgh. With prominent Indian academicians such as Rohan Ganguli, Mary Ganguli, and Keshavan generously offering their expertise and mentoring early career psychiatrists, it has been a nidus for many budding psychiatric researchers of Indian origin. Although often mentioned together, Rohan and Mary Ganguli have made very different and specialized contributions in their fields of expertise, respectively. Rohan Ganguli has conducted extensive studies in the immunology of schizophrenia, and in more recent years has concentrated his efforts on the metabolic burden in this disease, especially that associated with atypical antipsychotics. Mary Ganguli is a geriatric psychiatrist, best known for her work in the epidemiology and assessment of dementia, including a cross-national study between the Monongahela valley in Pennsylvania, USA, and Ballabgarh, Haryana, India. The nidus effect is also visible in many prominent universities where a researcher of Indian origin has excelled, attracting younger researchers to the department. Duke University is one such where Ranga Krishnan’s presence and leadership has led to a nexus of both senior and mid-level Indian psychiatrists, such as, Prakash Masand (psychosomatic medicine, psychopharmacology), Meera Narsimhan (Psychiatry and Primary Care, Telepsychiatry – now moved to the University of South Carolina as Research Vice-Chair), Ashvin Patkar (Addiction Psychiatry), Murali Doraiswamy (Brain Imaging, Biology of Mood Disorders), and others have gone to establish niche areas of research and onto academic leadership roles in their own right. Two other examples are (1) University of Texas, South Western Medical Center (UTSW), where MadhuKar Trivedi leads an internationally acclaimed mood disorders institute. Trivedi’s leadership role in the largest, publicly supported algorithmic treatment research program of depression, is well-recognized. This study (STAR-D) provides an excellent roadmap for treating depression for clinicians and researchers, alike. UTSW has 10 psychiatrists of Indian origin on its full-time faculty roster, with wide ranging academic specializations; (2) University of California at San Diego (UCSD) where Dilip Jeste has served as the magnet, with recent early-career psychiatric researchers Ipsit Vahia and Gowri Savla Nayak. These selective examples serve as powerful evidence of how brilliant individuals with research excellence transform programs and departments to the benefit of many. Institutions in India and elsewhere aspiring for their own excellence would be well advised to emulate this.

In reviewing the first wave of researchers, the work of Indo-Canadian investigators at McGill was mentioned. Within the second wave, the work of what might be termed the bipolar group is especially note worthy. Vivek Kusumakar at Dalhousie University, until he moved to Johnson and Johnson Company in the USA – he had an untimely death in 2009, and Lakshmi Yatham at University of British Columbia, Vancouver, both alumni of NIMHANS have lead major research in the treatment of bipolar disorders. They co-chaired the Canadian Network for Mood & Anxiety Treatments (CANMAT) and developed the bipolar treatment guidelines in Canada; Yatham is president of the International Society for Bipolar Disorders. The University of Western Ontario, University of British Columbia – Vancouver, University of Toronto and its affiliates have served as fertile grounds for the pursuit of academic careers by Indo-Canadian Psychiatrists (See table 1 for listing of individuals at these centres).

Table 1 lists 100 psychiatry researchers of Indian origin in USA and Canada. By no means is this tabulation a comprehensive list and during the research for this article, this author was amazed at the number of early to mid-career psychiatrists of Indian origin who are contributing to various subspecialties of psychiatry through their research. Nor is there any rigorous methodology in the selection. It is simply the assessment of one individual, with all its biases and short comings, albeit with the benefit of 35 years of observation.

In addition to those discussed earlier, from among the second wave of researchers, the contributions of the following individuals have also enriched our knowledge of psychiatric disorders and their treatment. In alphabetical order, Roy Chengappa (psychopharmacology of serious mental illnesses), D. Devanand (electroconvulsive therapy, memory disorders in the elderly), Sanjay Dube (psychopharmacology), Choudhary Jampala (phenomenology and biology of schizophrenia), Geeta Jayaram (safety and quality improvement), Shitij Kapur (biology of schizophrenia, brain imaging, and receptorology, now at King’s College and Institute of Psychiatry, London, UK), Arifulla Khan (psychopharmacology of depression, placebo effect), Vivek Kusumakar (mood and anxiety disorder treatment, child psychiatry), Ashok Malla (early psychosis), Anil Malhotra (genetics, pharmacogenomics), Rahul Manchanda (early psychoses, brain injury rehabilitation), Sukdeb Mukherjee (ECT, bipolar disorders), Viswajit Nimgaonkar (psychiatric genetics), Phillip Ninan (anxiety disorders, schizophrenia, Anand Pandurangi (structural and biological abnormalities in schizophrenia, psychopharmacology), Uma Rao (pediatric mood disorders and addictions), Rajiv Tandon (nosology of psychoses, psychopharmacology of psychosis), Gunwant Thaker (biology of schizophrenia), Lakshmi Yatham (bipolar disorder treatment), and Vikram Yeragani (anxiety disorders, heart rate variability).

Lest the reader get the impression that Indo-American psychiatric academicians have focused their careers only on biological psychiatry, it should be emphasized that many noteworthy contributions have been made in the psychological aspects as well [Table 2]. CV Ramana was an early psychoanalyst of Indian origin in the USA, and wrote an important article on the history of psychoanalysis in India. Dr. Salman Akhtar was among the first wave of researchers and was a leading psychoanalytic contributor from the USA. In fact, it would be no exaggeration to describe him as a world leader in this field. His studies on personality disorder, cultural aspects of the immigrant experience, and psychodynamics have earned him a marquee place in American psychodynamic psychiatry. Akhtar is a prodigious writer and multi-talented. He has published over 45 books on various aspects of psychodynamics. This brief article would not do any justice in documenting his productivity, and this author is not qualified to assess the significance of his extensive writings. Table 3 is dedicated to a listing of his books. Mantosh Dewan and colleagues have published, on brief psycho therapies, and the difficult to treat patient. Dewan’s study on the benefits of combined psychotherapy and medication management by the psychiatrist garnered national attention even as the profession of psychiatry (again) struggles with its identity. Prakash Desai at UIC has been a major contributor in the area of bioethics, especially from the perspective of its history in India.

**Table 2:** Indian–American psychiatrists — books published

Title	Author (S)/Editor (S) Publisher/Year
Anxiety disorders	
Contemporary diagnosis and management of anxiety disorders	Philip Ninan and Boadie W. Dunlop. Handbooks in Healthcare Company, 2006.
Bipolar disorder	
Bipolar disorder: A clinician’s guide to biological treatments	Lakshmi N. Yatham, Vivek Kusumakar *et al* 2^nd^ Edition. Routledge, 2009
Bipolar disorder: Clinical and neurobiological foundations	Lakshmi N. Yatham, Mario Maj John Wiley and Sons, 2010
Contemporary diagnosis and management of bipolar disorders	Samuel Gershon. K.N. Roy Chengappa Publisher: Assocs in Medical Marketing Co Inc, 2009
Clinical psychiatry	
Clinician’s guide to psychiatric care	Ranga Krishnan, Jane Gagliardi, Wei Jiang. Oxford University Press, 2008.
Psychiatry rounds: Practical solutions to clinical challenges	Nutan Atre-Vaidya. Medmaster, 2004
Psychiatric medicine: The psychiatrist’s guide to the treatment of common medical illnesses	Mahendra Dave, Kurt P Miceli, Poonam Modlia. Lippincott, Williams and Wilkins, 2008
Solving psychiatric puzzles	Vidyasagar Sethi. Pub: Authorhouse, 2004
Brain imaging in clinical psychiatry Chronic nonmalignant pain	K. Ranga Rama Krishnan and P. Murali, M.D. Doraiswamy Pub: M Dekker. 1997 Mathews M, Covington E. Cleveland Clinic Current Clinical Medicine, 2009, Elsevier Saunders.
Cognition - dementia	
Diagnostic issues in dementia: Advancing the research agenda for DSM-V The alzheimer’s action plan: What you need to know-and what you can do-about memory problems, from prevention to early intervention and care	Trey Sunderland, Dilip V. Jeste, Olusegun Baiyew, Appi, 2007 P. Murali Doraiswamy, Lisa P. Gwyther, and Tina Adler, St Martins Press, NY, 2009
The alzheimer’s action plan: The experts* guide to the best diagnosis and treatment	P. Murali Doraiswamy. Lisa P. Gwythe And Tina Adler. St Martin’s Press, NY 2008
Supporting the caregiver in dementia: A guide for healthcare professionals”	Sheila Loboprabhu. Victor Molinari, James Lomax. Johns Hopkins University Press, 2006
Culture	
Life in Color: culture in american psychiatry Overseas indians: A study in adaptation India in the United States: Contributions of India and Indians in the United States of America	Annelle Primm. Jambur Ananth. Pedro Ruiz, Rodrigo Munez. Hilton Pub, 2006 Ram P. Srivastava, George Kurian Stosius Inc/Advent Books Division, 1983 Sachin N. Pradhan. Sp Press International, Inc., 1996
Education and examination preparation	
Educational review manual in psychiatry EMQs for the PLAB part 1	K. Ranga Rama Krishnan. Pub: Caste Connonlly Graduate Medical, 2005 Mathews M, George V, Sebastian R. Science Publications Inc. New Hampshire. USA, 2001
Psychiatry: 1200 questions to help you pass the boards	Mathews M. Budur K. Basil B. Mathews M. Lippincott Williams and Wilkins, 2004
Multiple choice questions in psychiatry	Manchanda R. Churchill Livingstone. London. 1984
Ethics	
Medical ethics in India. In encyclopedia of the history of science, technology, and medicine in non-western cultures	Desai P Kluwer Academic Publishers Dordrecht, The Netherlands, 1997
History of medical ethics. South Asia. India. In the encyclopedia of bioethics	Desai P.: Mcmillan Publications. New York. 1995.
Triumph and tragedy: Psychohistorical decisions of Mahatma Gandhi	Desai P. And Muslin H Haranand. New Delhi, India, 1998
Miscellaneous	
Core evolving technologies in biomedical engineering	Sree Han Rao V, Sony M. Chokka P, Baer K, Yeragani Vk. Orient Longman Press
Disaster psychiatry	Anand Pandya. (With Craig Katz, Robert Coles) Analytic Press, 2005
Pharmacology and psychpharmacology	
Psychophannacology – treatment of psychiatric disorders	Jambur Ananth. Jaypee Brothers Medical Publishers (P) Ltd. 1999
Neuroleptic-induced movement disorders: A comprehensive survey	Dilip V Jeste. Ramzy Yassa. NPV Nair. Cambridge University Press. 1997
Neuroleptic-induced movement disorders	Ramzy Yassa. N. P. V. Nair And Dilip V. Jeste Cambridge University Press. 1997
Understanding and treating tardive dyskinesia	Dilip Jeste, Richard J Wyatt. The Guilford Press. 1982
Pharmacology in medicine – principles and practice	Sachin N. Pradhan. Sp Pr International. 1986
Psychotherapy (see separate table for the works of Sahnan Akhtar)	
The difficult-to-treat psychiatric patient	Mantosh Dewan, Ronald Pies. Appi 2001
The art and science of brief psycho therapies	Mantosh Dewan, E Steenbarger. Roger P Greenberg. APPI 2004
Schizophrenia	
Dopamine in the pathophysiology and treatment of schizophrenia	Shitij Kapur, Yves Lecrubier, Informa Healthcare. 2003
Schizophrenia	Mathews M. Muzina D. Cleveland Clinic Current Clinical Medicine. 2009. Elsevier Saunders
Secondary schizophrenia	Parminder Sachdev, Matecheri Keshavan; Cambridage University Press, 2009
Neurodevelopment and schizophrenia	Matched S. Keshavan, James L. Kennedy. Robin M. Murray. Cambridge University Press, UK, 2004
Clinical practical treatment guidelines — Schizophrenia	Murray W Enns, Laurence Katz. Ashok Malla. Michael Myers, Scott Patten, Gilbert Pinard. Bruce Pollock. Canadian psychiatric Association, 2005
**Women’s Mental Health**	
Shouldn’t I Be Happy: Emotional problems of pregnant and postpartum women	Shaila Misri. The Free Press, Simon and Sinister, NY 1985
Pregnancy Blues: What every woman needs to know about depression during Pregnancy	Shaila Kulkarni Misri. Bantam Dell. Random House Inc, NY. 2005
Women’s health and psychiatry	J.F. Rosenbaum, Kimberly H. Pearson Pearson, Shamsah B. Sonawalla. Lippincott Williams and Wilkins. 2002

**Table 3:** Books by Dr. Salman Akhtar

New Psychiatric Syndromes - DSM III and Beyond	Jason Aronson, 1984
The Hidden Knot	Adams Press, 1985
Beyond the Symbiotic Orbit: Advances in Separation-Individuation Theory: Essays in Honor of Selma Kramer, M.D. (Co-Ed Henri Parens)	Analytic Press, 1991
The Trauma of Transgression: Psychotherapy of Incest Victims (Co-Ed Selma Kramer)	Jason Aronson, 1991
When the Body Speaks: Psychological Meanings in Kinetic Clues (Co-Ed Selma Kramer)	Jason Aronson, 1992
Broken Structures: Severe Personality Disorders and their Treatment	Jason Aronson 1992
Quest for Answers: A Primer of Understanding and Treating Severe Personality Disorders	Jason Aronson, 1994
Mahler and Kohut: Perspectives on Development, Psychopathology, and Technique (Co-Ed Selma Kramer)	Jason Aronson, 1994
The Birth of Hatred (Co-Eds: Selma Kramer, Henri Parens)	Jason Aronson, 1995
The Internal Mother (Co-Eds: Selma Kramer, Henri Parens)	Jason Aronson, 1996
Intimacy and Infidelity (Co-Ed Selma Kramer)	Jason Aronson, 1996
The Seed of Madness (Co-Ed Vamik Volkan)	Int Universities Press, 1996
Intimacy and Infidelity: Separation Individuation Perspectives	Jason Aronson, 1996
Lacan Avec La Psychanalyse Americaine (with Michel Tort, Judith Feher Gurewich)	Denoeel, 1996
The Seasons of Life: Separation-Individuation Perspectives (Co-Ed: Selma Kramer)	Jason Aronson 1997
Turned to Light	Adams Press, 1998
Inner Torment	Jason Aronson, 1999
The Colors of Childhood (Co-Ed Selma Kramer)	Jason Aronson, 1998
Brothers and Sisters (with Selma Kramer)	Jason Aronson, 1999
Thicker Than Blood: Bonds Of Fantasy And Reality In Adoption (Co-Ed Selma Kramer)	Jason Aronson, 2000
Does God Help?, (Co-Ed Henri Parens)	Jason Aronson, 2001
New Clinical Realms	Jason Aronson, 2003
Mental Zoo (with Vamik Volkan)	International Universities Press, 2004
Objects Of Our Desire	Harmony Books, 2005
The Language of Emotions (with Harold P Blum)	Jason Aronson, Inc 2005
Cultural Zoo (Co-Ed Vamik Volkan)	International Press, Inc, 2005
Real and Imaginary Fathers(Co-Ed Henri Parens)	Jason Aronson, 2005
Freud Along the Ganges: Psychoanalytic Reflections on the People and Culture of India	W W Norton and Co Inc, 2005
Three Faces of Mourning: Melancholia, Manic Defense, and Moving on	Jason Aronson 2006
Interpersonal Boundaries: Variations and Violations	Jason Aronson, 2006
The Geography of Meanings: Psychoanalytic Perspectives on Place, Space, Land, and Dislocation (Co-Ed:M Teresa Savio Hooke)	Karnac Books, 2007
Regarding Others: Reviews, Responses, and Reflections	Pitchstone LLC, 2007
The Unbroken Soul: Tragedy, Trauma, and Human Resilience (Co-Ed: Henri Parens, Harold Blum)	Jason Aronson, 2008
The Crescent and the Couch: Cross-Currents between Islam and Psychoanalysis	Jason Aronson, 2008
The Wound of Mortality — Fear, Denial, and Acceptance of Death.	Jason Aronson, 2009
A Comprehensive Dictionary of Psychoanalysis	Karnac Books, 2009
Freud and the Far East: Psychoanalytic Perspectives on the People and Culture of China, Japan, and Korea	Jason Aronson, 2009
Turning Points in Dynamic Psychotherapy: Initial Assessment, Boundaries, Money, Disruptions, and Suicidal Crises	Karnac Books 2009
On Freud’s “The Future of an Illusion” (Co-Ed Mary Kay O’Neil)	Karnac Books 2009
The Damaged Core: Origins, Dynamics, Manifestations, and Treatment	Jason Aronson, 2009
Lying, Cheating, and Carrying on: Developmental, Clinical, and Sociocultural Aspects of Dishonesty and Deceit (Co-Ed Henri Parens).	Jason Aronson, 2009
Broken Structures — Severe Personality Disorders and their Treatment	Jason Aronson, 2010

There are three other important modalities of scholarly contributions made by psychiatrists of Indian origin in the USA and Canada. The first modality is through book writing or editing. Although sometimes a book is a culmination and summary of many years of an individual researcher’s work, it may also be a compilation of the research of many authors in one place, and at other times it is a more user-friendly, narrative of one’s experiences and observation of a subject in depth. Tables 2 and 3 lists the books authored or edited by Indian–American psychiatrists, as another index of their scholarly contribution. The second modality is primarily a service but, has added significantly to our knowledge of the cultural dimension of adaptation, mental health, and psychiatric disorders. The third modality is educational in nature. The second modality in this instance pertains to narrating, understanding, and assisting with the Indian immigrant experience and its attendant challenges, unique mental health issues, and cultural adaptation. Jambur Ananth and his son Karthik Ananth co-authored a book on the immigrant experience from the psychological perspective. A group of psychiatrists in New York City have also called attention to the cultural dimension in the treatment of the Indian–American patient. Although not a comprehensive list, this group includes Manoj Shah, Satish Verma, Ramanathan Viswanathan, Nalini Juthani, Ashvin Pandya, Seeth Vivek, and others. They have championed the cause of the first and second generation Indian immigrant, especially those with limited resources, to obtain specialized treatment tailored to their cultural needs. Indeed it is the foresight of several members of this group that culminated in the formation of the Indo-American Psychiatric Association (IAPA) in 1979. Their studies have been presented at National conferences and symposia and printed in professional publications.

The third modality has focused on the other dimension of academic psychiatry, namely, psychiatric education and has attracted some of the brightest minds among Indian–American psychiatrists. Mantosh Dewan was one of the earliest to chair a University Department of Psychiatry at the State University of New York in Syracuse (now re-named Medical University of Syracuse), followed by Ranga Krishnan at Duke University. Since then, and especially in the last decade, many Indian–American academicians have been appointed to this coveted leadership position including Nutan-Atre-Vaidya, Anand Kumar, and Murali Rao, in Chicago. Many psychiatry residency directorships are (have been) occupied by Indo-Americans, including Chetan Haldipur, Bhaskar Dave, Nyapati Rao, Nalini Juthani, and others. Among the recipients of the Nancy Roeske Certificate for Teaching Excellence awarded by the American Psychiatric Association (APA), is a long list of psychiatrists of Indian origin. Nyapati Rao has especially played a strong role in the development of the residency curriculum, and the newer methods of examination for the American Boards. Geeta Jayaram distinguished herself by becoming the first (and so far only) Indo-American psychiatrist to have been the Scientific Program Director of the Annual APA meetings for four years, and Dilip Jeste and Prakash Desai are the only Indo-American psychiatrists to have won the national elections to important positions within organized psychiatry. These accomplishments although not traditional research are mentioned, as the reader will no doubt appreciate the impact of such accomplishments on the training opportunities, including research training, and on the academic recruitment, career development, and productivity of younger Indian psychiatrists migrating to America.

Significant scholarly contributions have also been made in specialties such as child and adolescent psychiatry by Shashi Elongovan (foster care), Vivek Kusumar (mood disorders, psychopharmacology), Uma Rao (pediatric mood disorders, addictions), Aradhana Sood (psychopharmacology, ADHD, campus violence), and others. Women’s mental health has benefited from the dedication and research of several Indian psychiatrists including Shaila Misri at the University of Vancouver in Canada, Shamsa Sonawalla in Harvard, and Lilian Gonsalves at the Cleveland Clinic. Ashok Srinivasaraghavan (University of Illinois, Springfield) has been a leader in Law and Psychiatry. Asha Mishra (Virginia Commonwealth University, Richmond, Virginia – VCU) in Community Psychiatry, Ramakrishna Shenoy (VCU) and Jaisimha Rao (Ontario, Canada) in Developmental Disorders, and Chetan Haldipur (Upstate Medical University, NY) to the history of psychiatry, especially as it pertains to ancient India, to name only a few, have made unique contributions.

### Third Wave (1991-Now)

In the twenty-first century, a new and much larger group of academicians of Indian origin is emerging to carry on the good work of their predecessors. Of the 5000+ psychiatrists of Indian origin in North America, it is estimated by this author that over 500 are now in academic psychiatry. A small sampling of names is included in Table 1. Not only is this group pursuing the traditional areas of scholarship and teaching, they are also blazing the trail in newer areas such as tele-psychiatry, web psychiatry, genomics, community service models, global psychiatry, computational psychiatry, neural networks, disaster psychiatry, and so on.

There can be no question that Indian American psychiatrists have woven strong threads in the fabric of American Psychiatry and that this experience has been a mutually advantageous relationship. Indian–American psychiatrists form a strong and integral part of American psychiatry, and it is hard to imagine the latter without the former. The growth of the IAPA and recognition of its formidable strengths is one indicator of this core role and strength.

### Challenges and Opportunities

The challenges confronting psychiatrists in India wishing to migrate and pursue a research career are uphill requirements of the American licensure system, limited availability of mentors, lack of strong networking within the Indo–American academic community, extremely competitive nature of federal funding, and the occasional glass ceiling that one confronts in career advancement. From the perspective of contributions in India by the Indian-American psychiatric research community, we should add that the absence of clear and user-friendly mechanisms in India for academic collaborations, joint appointments, restrictions on overseas travel for Indian collaborators, restrictions on research with DNA and biological tissues, and limited research infrastructure in most Indian institutions, have all worked to limit such contributions. The potential is indeed unlimited, and the benefit to advancement of knowledge and the care of our patients is tremendous, if only we can effectively address these challenges, and harness the full potential of the proven current generation and the extremely talented next generation in both countries. The Indo-US and Global Health Care Summits and the recent Indo-Global Psychiatry Initiative are good examples of current efforts to do so. On balance though, the accomplishments have far outweighed the challenges. We may comfortably and confidently declare this natural experiment of immigration to the USA / Canada a colossal success for the psychiatrist, psychiatry, and most importantly our patients.
